# The Local Treatment of Laryngeal Tuberculosis

**Published:** 1884-10

**Authors:** 


### The Local Treatment of Laryngeal Tuberculosis.

Dr. Gouguenheim (*Bul. Gen. de Ther.*, May 30, 1884) lays down some general rules for the local treatment of tuberculosis of the larynx. He divides the treatment into: 1. The use of medicated solutions. 2. That of solid applications, or crayons. 3. That of the galvanic cautery. 4. Surgical operations—partial avulsion, tracheotomy, or extirpation of the larynx. The use of crayons he does not approve of, believing it to be more defective, less convenient, and more difficult to support than the use of fluid applications. He makes an exception of iodoform, but prefers to use even that on a small sponge with a holder. He gives the following general rules for the use of the galvanic cautery: 1. When dysphagia is due to increase in the size of the epiglottis, the weekly application of the cautery at a few points brings about a rapid and lasting benefit. 2. When dysphagia is due to swelling of the folds and of the arytenoid region, cauterization is more painful than in the former case, and is not followed by the same good results. 3. When the vestibule and superior vocal cords are involved, the cautery is well borne, but the effects are not so good as in the first case. 4. When the larynx is the seat of numerous vegetations, cauterization is absolutely indicated, and should always be preferred to avulsion. 5. When the inferior vocal cords are thickened and covered with vegetations, they may be cauterized with advantage, but only if they are movable and if there is no stenosis. 6. When the vocal cords are approximated, and cannot be separated, and stenosis exists either by spasm of the adductors or paralysis of the dilators, there is danger in carrying the caustics into the interior of the larynx; but this danger is less than that which follows fluid applications. As to the avulsion of vegetations, he believes it to be bad practice. It is painful, and creates wounds which may serve as points of inoculation. He much prefers the galvanic cautery. He enumerates the indications for tracheotomy as follows: 1. With a tuberculous patient, where the lesions in the lungs are not extensive, the general state is satisfactory, and the temperature nearly normal, when frequent attacks of suffocation are caused by the laryngeal stenosis. 2. The existence of extensive pulmonary disease does not contra-indicate the operation if the temperature is normal and the digestive functions are undisturbed.

Of the relief obtained by tracheotomy in this affection of the larynx, Dr. Morrell Mackenzie speaks in the following words: "If there is much dyspnœa, tracheotomy should be performed, but the effect of the operation is, as a rule, only to prolong a miserable existence. I cannot recommend the operation as in any sense curative, and quite agree with Dr. Solis Cohen, who remarks that 'it cannot be curative, either directly or indirectly, and is only justifiable to ward off asphyxia, from œdema, tumefaction or impaction of necrosed cartilage.' He goes on to say that 'in opposing tracheotomy in laryngeal phthisis, except when there is urgent dyspnœa, I differ entirely from my accomplished pupil, Dr. Beverly Robinson, who observes that in order to obtain these latter (*i. e.*, favorable results) it seems indicated not to delay the operation, but, rather, to perform it so soon as the nature of the disease is obvious and other means appear of no avail.'"

